# Tyson on Examination of Urine

**Published:** 1874-12

**Authors:** 


A GUIDE TO THE PRACTICAL EXAMINATION OF URINE. For the use of physicians and students. By James Tyson, M.D., Hospital Lecturer on Pathological Anatomy in the University of Pennsylvania, etc., etc. With a plate and numerous illustrations. Philadelphia: Lindsay & Blakiston. 1875; 12mo.; cloth; pp. 182. Price $1.50. Sold in Atlanta by J. W. Burke & Co.

In the onward progress of medical science the scope of our means of rational diagnosis is constantly widening. Active and enterprising practitioners of medicine are devoting more and more attention, year by year, to the more exact and demonstrative diagnostic methods. Of the sourcess of knowledge in this direction the chemical and microscopical investigation of the urine is a prominent one, and a concise, accurate and practical hand-book upon the subject can but prove acceptable to the profession. Such an one, we think, Dr. Tyson has given us in the little volume before us; and so believing, we heartily commend it to our readers. The illustrations are numerous and excellent, and add greatly to the usefulness of the volume.

